# Isolated Group A Streptococcal Psoas Abscess in an Immunocompetent Young Woman: A Rare Complication Following a Gynecological Procedure

**DOI:** 10.7759/cureus.108427

**Published:** 2026-05-07

**Authors:** Zuhair Chaudhry, Saher F Zaidi, Rachel Battersby, Jessica G Avila, Su Hlaing

**Affiliations:** 1 Medicine, Kern Medical Center, Bakersfield, USA; 2 Medicine, Western University of Health Sciences, Pomona, USA; 3 Infectious Disease, Kern Medical Center, Bakersfield, USA; 4 Family Medicine, Western University of Health Sciences, Pomona, USA; 5 Family Medicine, Clinica Sierra Vista/UCLA-Kern Medical Center, Bakersfield, USA; 6 Family Medicine, Kern Medical Center, Bakersfield, USA

**Keywords:** bacteremia, conservative management, group a streptococcus, iliopsoas myositis, immunocompetent host, invasive streptococcal infection, medication abortion, parameningeal reaction, psoas abscess, streptococcus pyogenes

## Abstract

Psoas abscess is a rare but serious infection most commonly caused by *Staphylococcus aureus* or enteric organisms. *Streptococcus pyogenes* is an exceptionally rare etiology, reported only in isolated cases. Invasive group A streptococcus (GAS) infections may follow gynecological procedures and can occur in otherwise healthy individuals. This report highlights an atypical presentation of GAS bacteremia with psoas myositis and developing abscess in an immunocompetent patient.

A 21-year-old previously healthy woman presented with a 10-day history of worsening bilateral lower-extremity pain, fever, and inability to ambulate, beginning two weeks after a medication-induced abortion. On presentation, she was febrile and tachycardic, with significant leukocytosis (32,000/μL) and elevated inflammatory markers. Given her severe pain and limited mobility, initial concern for neurologic pathology prompted a lumbar puncture, which demonstrated cerebrospinal fluid pleocytosis. Empiric therapy for meningitis was initiated. Blood cultures later grew *Streptococcus pyogenes*, confirming bacteremia, and antibiotics were narrowed accordingly. Neurologic evaluation ultimately deemed meningitis unlikely, with findings consistent with a parameningeal inflammatory response. CT of the abdomen and pelvis demonstrated inflammation of the psoas and iliacus muscles, with interval imaging showing a small developing psoas abscess that was not amenable to drainage. No alternative infectious source was identified. The patient was managed with targeted intravenous antibiotics and supportive care. She demonstrated progressive clinical improvement, regained independent ambulation, and was discharged on oral antibiotics with outpatient follow-up.

GAS-associated psoas abscess is exceedingly rare, particularly in immunocompetent patients without traditional risk factors. In this case, a recent medication-induced abortion likely served as the portal of entry through mucosal disruption and hematogenous spread. Diagnosis was complicated by an atypical presentation mimicking meningitis, including sterile cerebrospinal fluid pleocytosis. Recognizing that a parameningeal inflammatory response can be a secondary manifestation of a nearby infection is essential; this understanding encourages clinicians to look beyond the central nervous system and avoid delays in identifying retroperitoneal sources. Management depends on the abscess size and clinical stability. This report adds to evidence suggesting that small abscesses may be successfully managed with antibiotics alone. Additionally, the absence of localizing gynecologic symptoms may obscure the source of infection, necessitating a high index of suspicion.

This report demonstrates that *Streptococcus pyogenes* can cause deep musculoskeletal infection in healthy individuals and may present with misleading neurologic manifestations. Early recognition, appropriate imaging, and targeted therapy are critical. Clinicians should consider a deep soft-tissue infection in post-gynecologic patients presenting with severe pain and systemic symptoms, even in the absence of classic risk factors.

## Introduction

Psoas abscess is a rare but serious infection characterized by a purulent collection within the iliopsoas muscle compartment [1]. Although historically uncommon, its increasing incidence, from approximately 0.5 to 6.5 cases per 10,000 admissions, likely reflects improved imaging and clinical recognition [2]. Psoas abscesses are broadly categorized as primary, resulting from hematogenous spread of bacteria from distant sources, or secondary, arising from contiguous spread from adjacent gastrointestinal, genitourinary, or spinal sources. Methicillin-sensitive *Staphylococcus aureus* (MSSA) remains the most common causative organism in primary iliopsoas abscesses, while enteric organisms such as *Escherichia coli* predominate in secondary abscesses originating from gastrointestinal or genitourinary sources [3,4]. While *Staphylococcus aureus* and enteric pathogens account for the majority of cases, reports of Streptococcus pyogenes (Group A Streptococcus (GAS)) are exceptionally rare, and are almost always limited to isolated case reports, which makes its identification clinically significant [4,5,6].

One of the first documented primary GAS psoas abscesses was reported in 2003, highlighting the exceptional rarity of this organism within the already uncommon category of primary abscesses [5]. Subsequent reports have remained extremely limited [6]. This rarity contrasts with GAS’s well-established ability to cause aggressive invasive soft tissue infections. Notably, approximately 50% of invasive GAS infections occur without an identifiable portal of entry, often following minor or blunt trauma, and may progress rapidly to streptococcal toxic shock syndrome, which carries reported mortality rates of 38-45% [7]. Importantly, GAS bacteremia may occur following gynecological procedures and pregnancy-related events, including spontaneous pregnancy loss, medically or surgically induced abortion, and the postpartum period, providing a potential source for hematogenous seeding even in otherwise healthy individuals [7]. Recognition of these atypical sources of invasive GAS infection is essential, particularly when evaluating young, otherwise healthy patients of reproductive age presenting with deep soft tissue infections such as psoas abscess.

## Case presentation

A 21-year-old previously healthy woman with a recent medication-induced abortion presented with severe bilateral lower extremity pain, fevers, and inability to ambulate. She was ultimately found to have *Streptococcus pyogenes* bacteremia with suspected psoas myositis and a developing psoas abscess. The patient presented to the emergency department on November 11, 2025, and reported 10 days of bilateral lower extremity pain and swelling accompanied by fevers, beginning approximately two weeks after a medication-induced abortion and associated vaginal bleeding. On presentation to the emergency department, she was febrile (temperature 39.3 °C) and tachycardic (heart rate 114 bpm). She also had extreme pain that significantly limited her mobility, but did not display any focal neurologic deficits. Laboratory analysis demonstrated leukocytosis of 32,000/μL, an elevated erythrocyte sedimentation rate of 88 mm/hr, and an elevated C-reactive protein level of 12.00 mg/dL.

Given her limited mobility and concern for possible neurological involvement, a lumbar puncture was performed, revealing cerebrospinal fluid with a white blood cell count of 131 cells/μL and normal protein and glucose levels. Empiric broad-spectrum therapy for suspected meningitis was initiated with intravenous vancomycin, acyclovir, and fluconazole. Oral azithromycin was additionally initiated due to concern for a possible sexually transmitted infection, including pelvic inflammatory disease. Subsequent blood cultures grew *Streptococcus pyogenes* (GAS), and the patient was diagnosed with sepsis. Intravenous ceftriaxone was continued, while vancomycin, fluconazole, acyclovir, and azithromycin were discontinued following Infectious Disease consultation, as meningitis and pelvic inflammatory disease had been ruled out.

**Figure 1:**
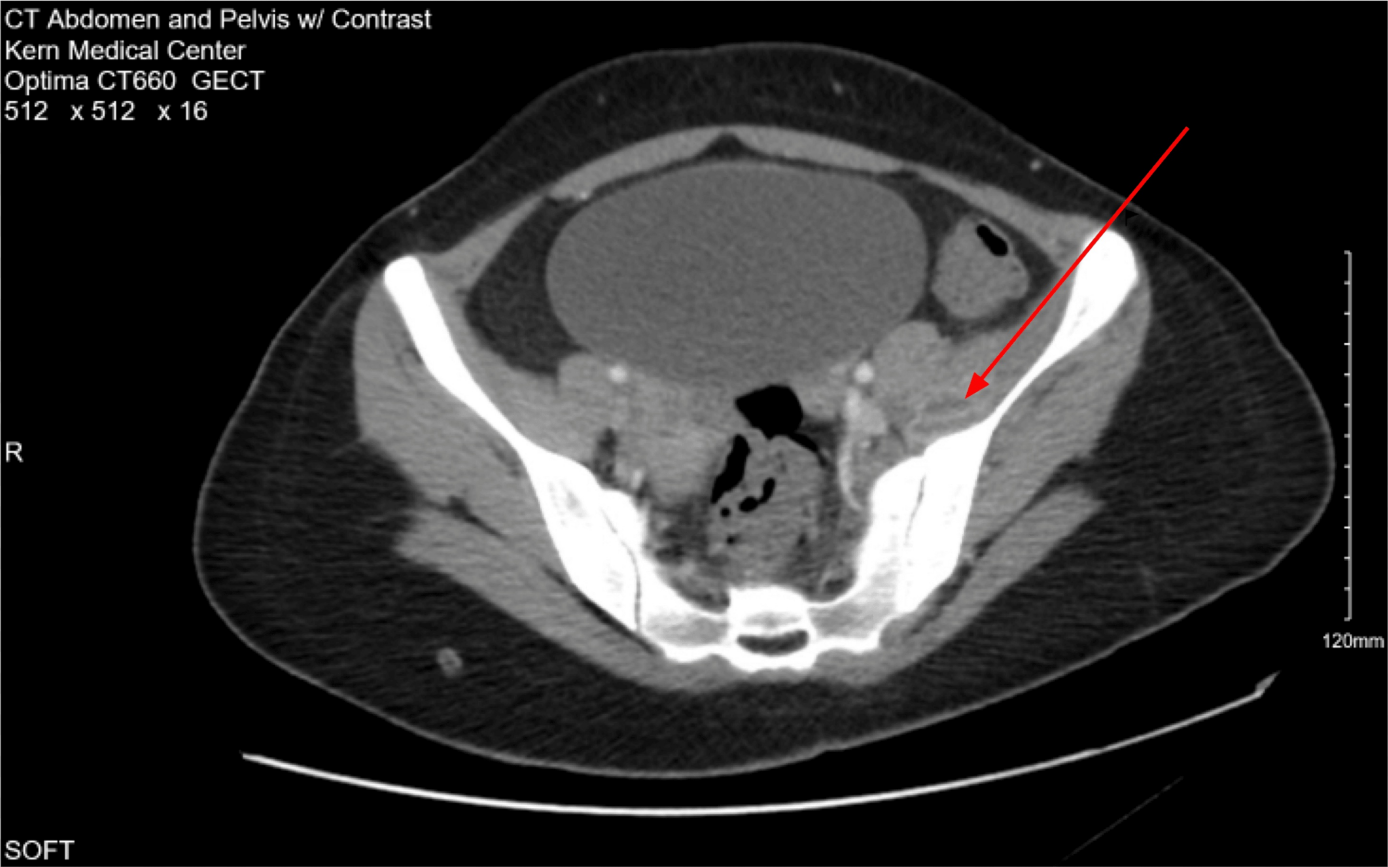
Axial contrast-enhanced CT scan of the abdomen and pelvis demonstrating a left-sided psoas abscess (arrow) CT: computed tomography

**Figure 2:**
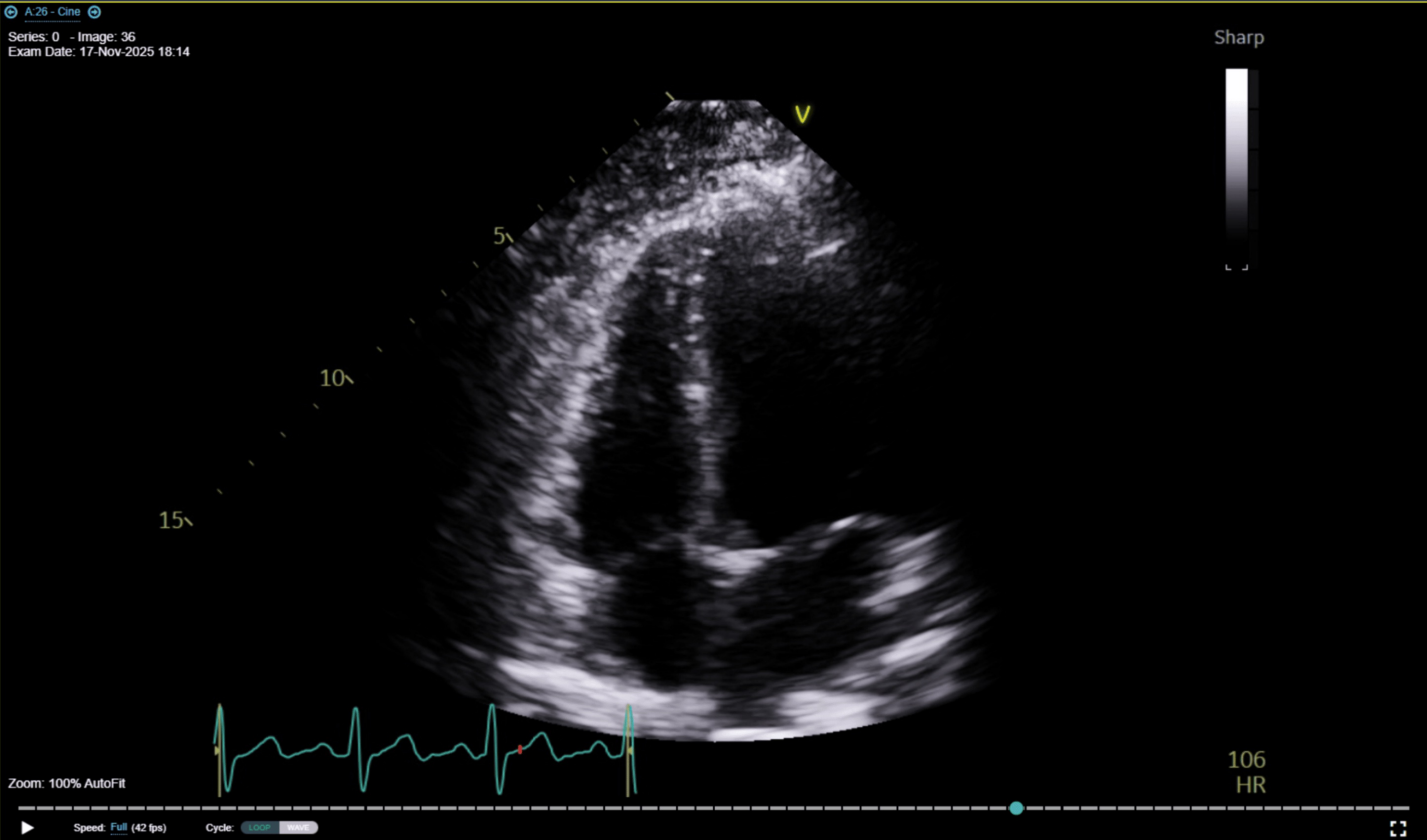
Transthoracic echocardiogram (apical four-chamber view) demonstrating no evidence of endocarditis or valvular vegetations

**Figure 3:**
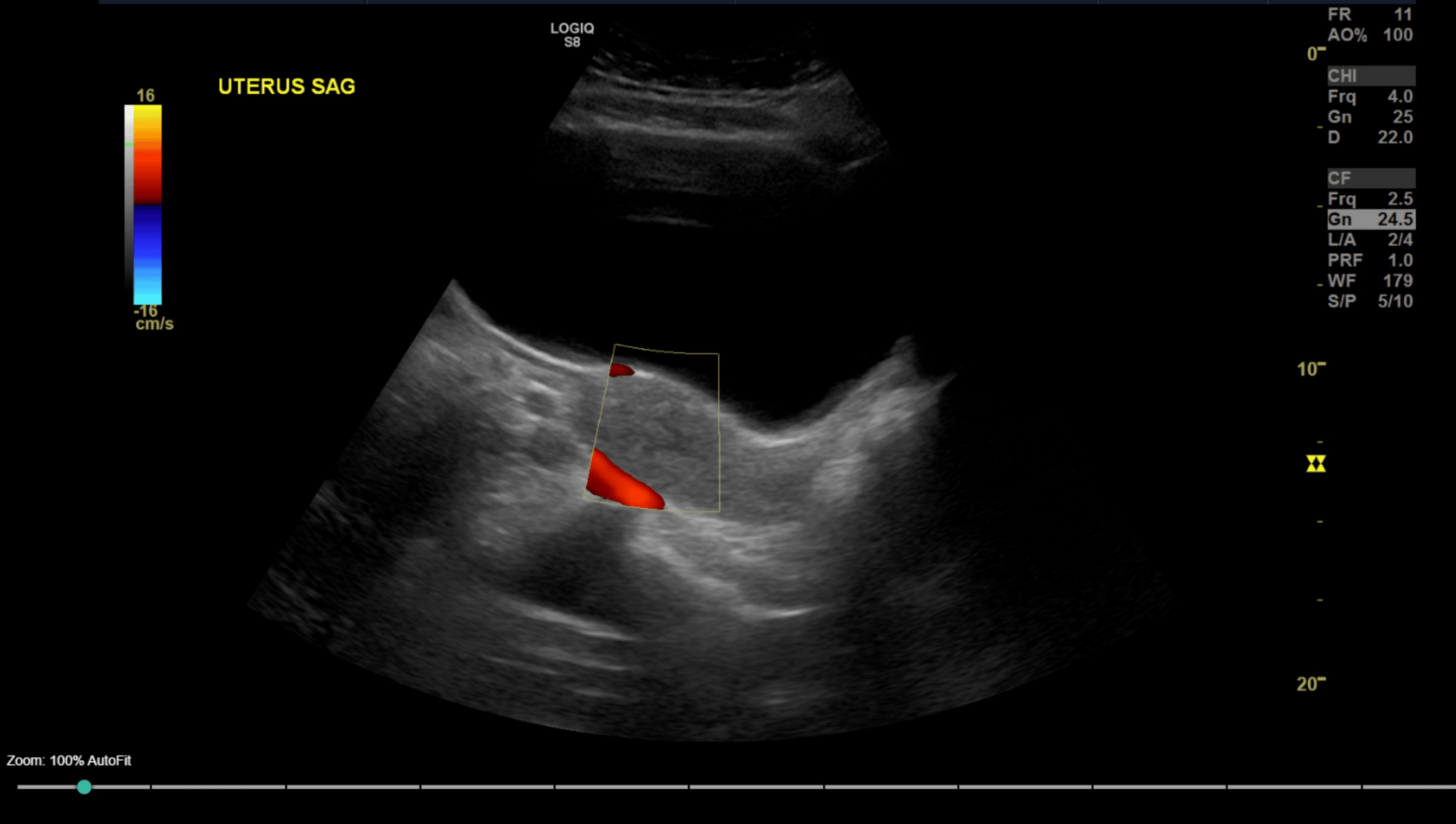
Sagittal ultrasound of the uterus demonstrating no evidence of retained products of conception

After consultation with the Department of Neurology, it was also determined that meningitis or myelitis was unlikely given the lack of clinical and laboratory evidence, and Neurology therefore reaffirmed the discontinuation of meningitis-directed therapy. On day three of hospital care (November 14, 2025), a repeat lumbar puncture was performed to further assess the underlying etiology, which showed marked improvement in cerebrospinal fluid pleocytosis, with 16 WBC/μL, further suggesting resolution of central nervous system inflammation and the absence of active meningitis. Of note, lumbar punctures in this patient were difficult due to dry taps and the patient’s limited pain tolerance. CT of the abdomen and pelvis completed on November 12, 2025, showed inflammation involving the left psoas and iliacus muscles (Figure 1). Close radiographic observation was recommended. Additionally, ultrasonographic evaluation showed no retained products of conception within the pelvis, and echocardiography revealed no signs of endocarditis (Figures 2, 3).

**Figure 4:**
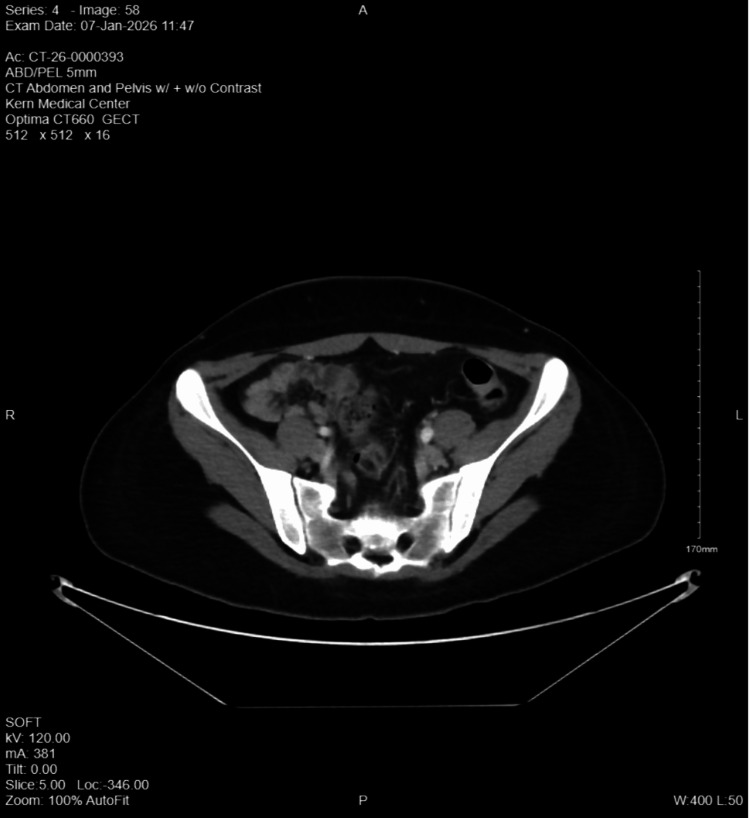
Follow-up CT scan of the abdomen and pelvis (January 7, 2026) demonstrating complete resolution with no evidence of a residual psoas abscess CT: computed tomography

The patient's wrist swelling and polyarthralgias improved markedly following antibiotic therapy. Progressive functional recovery was also observed with physical therapy. By November 18, 2025, she had gradually regained the ability to ambulate independently with minimal residual pain. Hemoglobin levels rose spontaneously to 9.3 g/dL without the need for transfusion. On the day of discharge, the patient remained hemodynamically stable, afebrile, and capable of performing activities of daily living independently. She reported mild abdominal discomfort attributed to gaseous distention. She denied fevers, chills, paresthesias, or weakness. She was prescribed oral amoxicillin 1 g three times daily for a six-week course. Repeat imaging obtained on January 7, 2026, demonstrated resolution of the psoas abscess, and the patient reported substantial improvement in ambulation (Figure 4, Table 1).

**Table 1:** Clinical laboratory findings at initial presentation and post-treatment follow-up The findings demonstrate the resolution of leukocytosis and the normalization of inflammatory markers

Parameter	Value (initial)	Value (January 5, 2026)	Reference range
Sodium, mmol/L	138	142	136-145
Potassium, mmol/L	3.8	3.7	3.5-5.1
Chloride, mmol/L	108	109	98-107
CO_2_, mmol/L	22	27	21-32
Calcium, mg/dL	8.7	8.6	8.5-10.1
Blood urea nitrogen* (*BUN), mg/dL	10	12	7-18
Glucose, mg/dL	123	99	70-140
Creatinine, mg/dL	0.44	0.57	0.51-0.95
Albumin, g/dL	2.4	3.9	3.4-5.0
Alkaline phosphatase (ALP), unit/L	254	58	45-117
Alanine aminotransferase (ALT), unit/L	108	24	13-61
Aspartate aminotransferase (AST), unit/L	62	11	15-37
Bilirubin total, mg/dL	1.1	0.4	0.0-1.0
Magnesium, mg/dL	2.3	2.2	1.8-2.4
Phosphorus, mg/dL	4.0	3.5	2.5- 4.9
White blood cell count (WBC), x10^3^/mcL	32.3	9.7	4.5 - 11.0
Red blood cell count (RBC), x10^6^/mcL	3.44	3.97	3.75-5.20
Hemoglobin (Hgb), g/dL	9.5	10.4	11.1-15.4
Hematocrit (Hct), %	29.4	32.8	33.0-45.5
Mean corpuscular volume (MCV), fL	85.3	82.6	75.7-97.6
Platelets, x10^3^/mcL	389	390	150-450
C-reactive protein (CRP), mg/dL	12.00		<0.30
Erythrocyte sedimentation rate (ESR) Westergren, mm/hr	88	29	≤20
HCG, beta quantitative, m IntlUnit/mL	28		≤3

## Discussion

Isolated psoas abscess caused by *Streptococcus pyogenes* (GAS) is an extremely uncommon condition, with only isolated case reports described in the literature compared with the more common etiologies of *Staphylococcus aureus* or *Escherichia coli* [4]. Psoas abscesses classically present with nonspecific symptoms, including fever, back or flank pain, and impaired mobility, often resulting in delayed diagnosis with increased morbidity and mortality [8]. Reported risk factors commonly include diabetes mellitus, renal failure, malignancy, intravenous drug use, and immunocompromised states, none of which were identified in our patient, further highlighting the unusual nature of this presentation [8].

In this case, the recent medication-induced abortion likely represented the portal of entry for invasive GAS infection, consistent with prior reports in the literature. Disruption of the epithelial and mucosal barrier has been associated with invasive GAS disease and may permit hematogenous dissemination even in otherwise healthy individuals [7,9]. Although GAS most commonly colonizes the oropharynx and skin, experimental and clinical evidence demonstrate that *Streptococcus pyogenes* expresses surface proteins that facilitate colonization and persistence within the female genital tract, providing biological plausibility for genital mucosal carriage before invasive disease [10].

Primary psoas abscesses caused by GAS are rare but have been described and are thought to result from hematogenous spread in the absence of contiguous infection [5,6]. In this case, no alternative infectious focus was identified, and the patient’s denial of oral intercourse made oropharyngeal transmission less likely, further supporting a gynecologic source. The organism’s well-recognized virulence factors, including M protein-mediated immune evasion and exotoxin production, may explain the degree of systemic toxicity observed despite a relatively localized deep soft tissue infection [7].

Diagnostic evaluation was further complicated by the atypical presentation in this patient, which raised initial concern for neurologic pathology. The patient’s lack of focal findings on early examination, coupled with severe systemic toxicity and limited mobility, mimicked meningitis. This was further complicated by sterile cerebrospinal fluid pleocytosis, a phenomenon consistent with a parameningeal reaction where paraspinal inflammation irritates the meninges without direct bacterial invasion of the central nervous system. Recognizing this "meningitis mimic" is crucial, as it may distract clinicians from identifying the true source of infection in the retroperitoneal space. Furthermore, the absence of gynecologic symptoms, pelvic tenderness, or retained products of conception initially obscured the gynecologic source of the infection [7].

Management strategies for psoas abscesses typically depend on abscess size and patient stability. Although percutaneous drainage is the standard of care for larger fluid collections, this case supports existing guidelines suggesting that small abscesses (<3.5 cm) can be successfully managed with targeted antibiotic therapy alone [11]. In this case, initial conservative management was pursued due to the abscess size and clinical considerations. Ultimately, this case emphasizes that clinicians must maintain a high index of suspicion for deep soft tissue infections in post-procedural gynecologic patients, even when typical risk factors are absent and localizing signs are initially obscure [7]. 

A review of the existing literature reveals that GAS-associated psoas abscesses are not only rare but also frequently present with atypical features, including severe systemic illness, misleading neurologic signs, and the absence of traditional risk factors [4,5,6,8]. This case report contributes to the limited body of evidence by demonstrating a unique association with medication-induced abortion, a misleading meningeal presentation, and clinical improvement with initial conservative management despite radiographic progression [5,6,11]. However, Several limitations should be acknowledged. In our patient, causality between the gynecologic event and infection cannot be definitively established, and the proposed portal of entry remains presumptive in the absence of direct uterine cultures [7,9]. Additionally, studies on conservative management of psoas abscesses primarily report short-term outcomes with limited long-term follow-up or recurrence data, leaving room for further evaluation of long-term outcomes [11]. 

This report highlights the need for heightened clinical suspicion for deep retroperitoneal infections in post-procedural gynecologic patients, even in the absence of classic clinical signs or established risk factors [7,8]. Early recognition of atypical pathogens, awareness of meningeal mimics, and the use of individualized management strategies informed by both clinical and radiographic findings may be important in optimizing outcomes in rare presentations such as this case [4,11].

## Conclusions

This report highlights that *Streptococcus pyogenes* can cause deep musculoskeletal infections, including psoas myositis and abscess formation, even in young, immunocompetent patients. Psoas pathology may present primarily with severe lower-extremity pain and can mimic neurologic disease, resulting in delayed diagnosis. Early recognition of deep musculoskeletal infection, prompt imaging, and multidisciplinary management are essential for achieving favorable clinical outcomes.
